# Factors Influencing Adolescent Anxiety: The Roles of Mothers, Teachers and Peers

**DOI:** 10.3390/ijerph182413234

**Published:** 2021-12-15

**Authors:** Xin Chen, Mengge Li, Huoliang Gong, Zekun Zhang, Wei Wang

**Affiliations:** School of Psychology, Henan University, Kaifeng 475001, China; chenxinpsy@163.com (X.C.); ghl1976@163.com (H.G.); zzkpsy@163.com (Z.Z.); sea_wangwei@163.com (W.W.)

**Keywords:** adult attachment, psychological flexibility, adolescent anxiety, teacher support, peer support

## Abstract

Grounded in social–ecological system theory, the present study tested the mediating effects of maternal psychological flexibility and mother–adolescent attachment on the relationship between maternal adult attachment and adolescent anxiety as well as the moderating effects of teacher support and peer support on the relationship between mother–adolescent attachment and adolescent anxiety. In total, 1139 Chinese mothers and adolescents completed a set of questionnaires, including the Experiences in Close Relationships Scale, Parental Psychological Flexibility Questionnaire, Inventory of Parent and Peer Attachment, Trait Anxiety Inventory, and Multidimensional Scale of Perceived Social Support. The results revealed that maternal adult attachment had a positive impact on adolescent anxiety. The relationship between maternal adult attachment and adolescent anxiety was chain mediated by maternal psychological flexibility and mother–adolescent attachment. In addition, teacher support and peer support had moderating effects on the relationship between mother–adolescent attachment and adolescent anxiety. These findings support the systematic social ecosystem perspective and highlight the differences in the effects of different maternal adult attachment styles, teacher support, and peer support on adolescent anxiety.

## 1. Introduction

Anxiety is a common psychological health problem [1]. Adolescents with anxiety often experience other adaptation problems during adolescence, such as pathological internet use [2], sleeping problems [3], substance abuse [4,5], and poor academic performance [6]. Adolescents suffer from trait anxiety, which means that they remain in an anxious mood for long periods of time [7]. According to the microsystem concept in social–ecological systems theory, family and school play important mutually interacting roles through which they jointly affect adolescent mental health [8]. Additionally, Kim et al. [9] suggested that mothers play an important role in adolescent anxiety. Teachers and peers can also play roles in adolescent anxiety [10,11]. In addition to mothers, teachers and peers will have an impact on adolescents’ anxiety. At present, no study has incorporated mothers, teachers, and peers into the same model to investigate the influencing factors of adolescents’ anxiety. Therefore, based on the theory of social ecosystems, this study examines the influencing factors of mothers, teachers, and peers on adolescents’ anxiety from a two-way perspective of mothers and adolescents.

### 1.1. Maternal Adult Attachment and Adolescent Anxiety

Hazan and Shaver [12] extended attachment theory to the adult stage by suggesting that emotional connection to a partner in a romantic relationship can be regarded as a type of attachment relationship, i.e., adult attachment. The classification of adult attachment differs from the previous classification of individual attachment as secure attachment, avoidant attachment, and anxious attachment [13]. Brennan et al. [14] proposed the division of attachment into the following two continuous dimensions: attachment anxiety and attachment avoidance. Based on this view, numerous studies have used the Experiences in Close Relationships Scale [15,16] and found that individuals’ attachment anxiety and attachment avoidance are important personal physical and mental predictors of health and interpersonal relationship quality [17,18,19]. In the field of parenting, mothers with avoidant adult attachment may show more indifference and rejection in parenting and not be sensitive to the needs of their adolescents, resulting in their children lacking warm and effective support in stressful situations. Mothers with anxious adult attachment adopt more controlling and emotionally unstable interactions in their parenting behaviors and are unable to provide warm and effective support to their adolescents in stressful situations [20,21]. Mothers with secure adult attachment provide emotional stability and warm support when raising their children [22]. Warm and effective maternal support can provide a secure base for adolescents to explore the environment bravely in a non-stressful situation. In addition, such support provides a safe haven for adolescents under pressure [23] and reduces anxiety when adolescents face stressful situations [24].

### 1.2. Maternal Psychological Flexibility as a Mediator

Family system theory asserts that a family can be divided into different hierarchical “energy” subsystems, such as the marriage subsystem and the parent–child subsystem [25]. The energy generated in one subsystem can be directly transferred to another subsystem, implying that family subsystems are mutually influential [26]. For example, the marriage subsystem represents the characteristics and functions of the interactions between a husband and wife in their roles as a couple, and the mother–child subsystem represents the characteristics and functions of the interactions between a mother and her child. Maternal psychological flexibility refers to the ability of parents to accept their negative thoughts, emotions, and impulses toward their adolescents and still maintain effective parenting behaviors during the parenting process. Therefore, maternal adult attachment, which occurs within the marital subsystem, affects maternal psychological flexibility, which occurs within the mother–child subsystem. Crowell and Feldman [26] found that mothers with higher adult attachment avoidance and anxiety are more likely to use rigid and inflexible parenting behaviors while raising their adolescents [27,28] such that adolescents with rapid and unbalanced physical and mental development do not receive flexible and effective responses and support when they encounter stressful situations, which aggravates their anxiety in stressful situations.

### 1.3. Mother–Adolescent Attachment as a Mediator

Mother–adolescent attachment refers to the lasting and strong emotional bond established between mothers and adolescents [29,30]. According to the overflow hypothesis of family system theory, maternal adult attachment in the marriage subsystem overflows to affect mother–adolescent attachment in the mother–child subsystem [31]. Mothers with avoidant adult attachment habitually adopt evasive and indifferent responses when interacting with their adolescents. These adolescents believe that their mothers cannot provide a safe haven; thus, they form an unsafe mother–adolescent attachment with their mothers [32]. Mothers with anxious adult attachment are more likely to adopt controlling and unstable response methods when interacting with their adolescents. These mothers are also unable to provide a stable, secure safe haven for their adolescents; thus, an unsafe mother–adolescent attachment is formed [33]. As noted by Bowlby [34], individuals with safe mother–adolescent attachment report that their parents provide a safe haven that allows them to relieve their anxiety when they encounter stressful situations. The safer the mother–adolescent attachment is, the more psychological support the adolescent can obtain when faced with stressful situations. Moreover, the stronger an individual’s ability to resist anxiety, the less likely he or she is to suffer its adverse effects [35,36]. Empirical studies also show that good mother–adolescent attachment quality can effectively reduce adolescent anxiety [37,38].

### 1.4. Maternal Psychological Flexibility and Mother–Adolescent Attachment as a Chain Mediator

Fu et al. [39] showed that a significant positive correlation exists between maternal psychological flexibility and mother–adolescent attachment. The higher the maternal psychological flexibility is, the more sensitive and flexible a mother is to the psychological needs of her adolescent, and the more effective she is in responding to her child’s psychological needs; inflexible and rigid parenting behavior cannot provide flexible and effective support to adolescents [40]. When adolescents are handling stressful situations, mothers with high psychological flexibility in their parenting can effectively provide them with a safe haven [41]. Effectively providing a safe haven to adolescents can promote the formation of secure mother–adolescent attachment between mothers and adolescents [42].

### 1.5. Teacher Support and Peer Support as Moderators

According to the microsystem concept in Bronfenbrenner and Ceci’s social–ecological systems theory of individual development [43], families and schools both represent systems that have an important influence on individual growth. Teachers and peers are individuals who, in addition to parents, have important influences on adolescents [44]. Research has shown that teacher support and peer support directly promote the psychological development of adolescents [45,46]. The microsystem concept in social–ecological systems theory emphasizes that the interaction between different systems affects adolescents’ growth, and this concept has been used to explore how the family (mother–adolescent attachment) and school (teacher support and peer support) interact to affect adolescents’ anxiety; such exploration is important for reducing adolescent anxiety [47,48]. In the previous literature, research concerning the joint effects of teacher support, peer support, and family on the mechanism of adolescent anxiety is lacking. Studies have shown that high teacher support can promote the effect of parent–child attachment on adolescent anxiety [49], suggesting that with adequate social support, adolescents are more likely to feel warmth and support from their mothers and that high teacher support and peer support may promote the reducing effect of mother–adolescent attachment on adolescent anxiety.

Using mother–adolescent pairs as the research objects and drawing upon social–ecological systems theory and family systems theory, this study integrates family and school factors into one model to explore their impacts on adolescent anxiety. It expands the previous literature by simultaneously examining the chain-mediating effects of maternal psychological flexibility and mother–adolescent attachment on the relationship between maternal adult attachment and adolescent anxiety. Moreover, the moderating effects of teacher support and peer support on the relationship between mother–adolescent attachment and adolescent anxiety are examined. Drawing upon the above evidence, we developed the following hypotheses:

**Hypothesis** **1.** *Maternal adult attachment is positively related to adolescent anxiety.*

**Hypothesis** **2.** *Maternal psychological flexibility mediates the relationship between maternal adult attachment and adolescent anxiety.*

**Hypothesis** **3.** *Mother–adolescent attachment mediates the relationship between maternal adult attachment and adolescent anxiety.*

**Hypothesis** **4.** *Maternal psychological flexibility and mother–adolescent attachment play a chain-mediating role in the relationship between maternal adult attachment and adolescent anxiety.*

**Figure 1:**
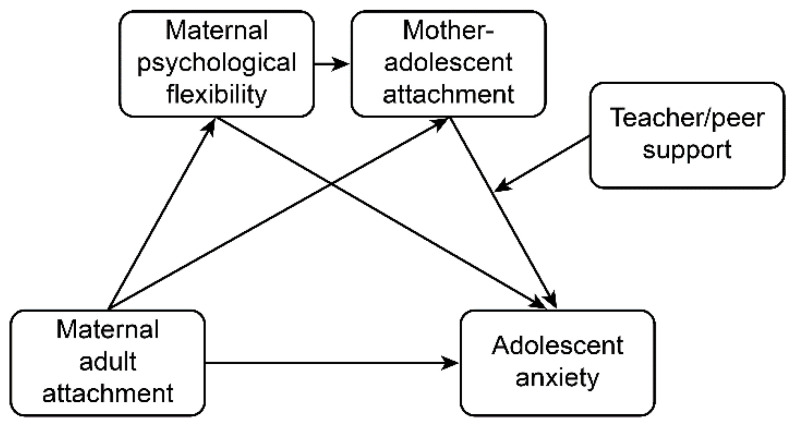
Diagram of the hypotheses and model.

**Hypothesis** **5.** *Teacher support and peer support play moderating roles in the relationship between mother–child attachment and adolescent anxiety. A diagram of the model is shown in Figure 1.*

## 2. Materials and Methods

### 2.1. Participants and Procedures

#### 2.1.1. Participants

A total of 1139 mother–adolescent pairs were involved in the study. All the participants in the present study were mothers and focal adolescent offspring from Chinese families. In Chinese society, mothers are usually the main nurturers of adolescents. In order to explore the influence of mothers on adolescents’ anxiety more clearly, this study includes only mothers and adolescents as research subjects [23]; therefore, fathers were not selected for the study. The age range of the teenagers was 10 to 16. The mean age of the adolescents was 12.042 years (*SD* = 1.278). Studies have shown that adolescents aged 10–16 years face an elevated risk of anxiety problems, and studying the causes of adolescent anxiety during this period can help reduce adolescent anxiety [49,50,51]. In addition, 52% of the adolescents were boys and 48% were girls. The mean age of the mothers was 40.513 years (*SD* = 6.238). Regarding educational attainment among the mothers, 24.8% had progressed as far as primary school, 62.9% were middle school graduates, 5.9% were high school graduates, and 6.5% held college degrees or above. All the mothers were married or had previously been married; 49 of the mothers were divorced at the time of the study.

#### 2.1.2. Procedures

The present study used a convenience sampling method with a group of families, including adolescents studying in either primary or secondary school and their mothers. The study was approved by the Research Ethical Committee of the author’s university. All the participating mothers and adolescents provided written informed consent. The participating students and mothers were free to withdraw from the study at any time. The adolescents completed questionnaires in class; they provided their demographic information, including their gender and age, and subsequently completed the remaining measures, which assessed their attachment to their mothers, their anxiety, and their peer and teacher support. Furthermore, the adolescents brought home questionnaires to be filled out by their mothers. Each mother completed measures assessing her adult attachment to her spouse and her parental psychological flexibility. The adolescents brought the mothers’ completed questionnaires back to school.

### 2.2. Measurements

#### 2.2.1. Mother-Reported Adult Attachment

The Experiences in Close Relationship Scale (ECR) revised by Li and Kato [16] (ECR-R) was used to examine adult attachment. The ECR-R is a 36-item, self-report questionnaire that assesses anxious and avoidant attachment and measures attachment anxiety (e.g., “I worry about being abandoned”), and attachment avoidance (e.g., “I prefer not to show a partner how I feel deep down”). The participants responded to the questions on a 7-point Likert scale ranging from 1 (strongly disagree) to 7 (strongly agree); higher scores indicated higher attachment anxiety or avoidance. The reported Cronbach’s alphas were 0.805 for the anxiety subscale and 0.910 for the avoidance subscale.

#### 2.2.2. Mother-Reported Parental Psychological Flexibility Questionnaire

The Parental Psychological Flexibility Questionnaire (PPFQ) was developed by Burke and Moore [52] and revised by Li et al. [53]; the PPFQ has 16 items covering the following three dimensions: cognitive dissociation (e.g., “My worries prevented me from being a good mother”), commitment to action (e.g., “I can be a good mother even if I’m mad at my child”), and acceptance (e.g., “The unpredictability of parenting makes parenting fun and rewarding”). The participants responded to the questions on a 7-point Likert scale ranging from 1 (strongly disagree) to 7 (strongly agree); some questions used a reverse scoring method. Higher total scores indicated higher parental psychological flexibility. The reported Cronbach’s alpha was 0.841 in this study.

#### 2.2.3. Adolescent-Reported Mother–Adolescent Attachment

We adopted items related to mother–adolescent attachment selected from the Inventory of Parent and Peer Attachment, which was developed by Armsden and Greenberg [54] and revised by Li et al. [55], with 13 items covering the following three dimensions: trust (e.g., “Dad respected my feelings”), communication (e.g., “Dad helped me to discuss my difficulties”), and alienation (e.g., “I was angry with my dad”). The participants responded to the questions on a 5-point Likert scale ranging from 1 (strongly disagree) to 5 (strongly agree); higher scores indicated higher mother–adolescent attachment. The reported Cronbach’s alpha was 0.843 in this study.

#### 2.2.4. Adolescent-Reported Trait Anxiety

We adopted the Trait Anxiety Inventory from the State-Trait Anxiety Inventory (STAI), which was developed by [56] and revised by Li and Qian [57]. The scale has 20 items (e.g., “I worry too much about things that don’t really matter”). The participants responded to the questions on a 4-point Likert scale ranging from 1 (little or none of the time) to 4 (most or all of the time); higher scores indicated higher trait anxiety. The reported Cronbach’s alpha was 0.843 in this study.

#### 2.2.5. Adolescent-Reported Teacher Support and Peer Support

We adopted items related to teacher support and peer support selected from the Multidimensional Scale of Perceived Social Support (MSPSS), which was developed by Zimet et al. [58] and revised by Zhao and Li [59]; the scale has 8 items and the following two dimensions: teacher support (4 items) (e.g., “Teachers care about my feelings in my life”) and peer support (4 items) (e.g., “My friends can really help me”). The participants responded to the questions on a 5-point Likert scale ranging from 1 (strongly disagree) to 5 (strongly agree); higher scores indicated higher teacher support and peer support. The Cronbach’s alpha for teacher support and peer support were 0.907 and 0.870, respectively, in this study.

### 2.3. Data Analysis

To test the hypotheses, a composite scale variable was generated in SPSS 23 and analyzed in the SPSS PROCESS macro version v3.4, as suggested by Hayes [60]. The advantage of using the PROCESS macro is that it generates an index of moderated mediation with simple slope results (standard error, t value, *p* value), providing an improved understanding of the relationship between variables. For each of the five hypotheses of the study, we calculated the 95 percent confidence interval by bootstrapping with 5000 iterations. The 95% confidence interval (CI) for the indirect effect was a bias-corrected estimate based on 5000 bootstrapping resamples. The mediating and moderating effects were considered significant at the level of *p* < 0.05. The pattern of the missing values was analyzed and recognized as completely random. Missing data were handled with listwise deletion [61].

## 3. Results

### 3.1. Preliminary Analysis

The means, standard deviations, and correlations of all the variables are presented in Table 1. Maternal adult attachment avoidance and anxiety are positively related to adolescent anxiety. Maternal psychological flexibility and mother–adolescent attachment are negatively related to adolescent anxiety. Maternal adult attachment anxiety and avoidance are negatively related to maternal psychological flexibility and mother–adolescent attachment. Age is positively related to adolescent anxiety, mother’s education is negatively related to adolescent anxiety, and gender is not related to adolescent anxiety.

### 3.2. Testing the Indirect Effect of Parent–Adolescent Attachment

Using model 6 of the SPSS PROCESS macro compiled by Hayes et al. [50], we repeated the extractions 5000 times. Because the mother’s education level and the adolescent’s age are related to adolescent anxiety, we controlled for age and mother’s education, and we tested the chain-mediating effects of maternal psychological flexibility and mother–adolescent attachment on maternal adult attachment and adolescent anxiety.

The results are shown in Table 2. The total indirect effect of the chain-mediating effect of maternal psychological flexibility and mother–adolescent attachment on the relationship between maternal adult attachment avoidance and adolescent anxiety is 0.094, accounting for 60% of the total effect. The confidence interval does not contain 0, indicating that maternal psychological flexibility and mother–adolescent attachment have a significant chain–mediating effect on the relationship between maternal adult attachment avoidance and adolescent anxiety.

The chained mediation contains the following three paths: maternal adult attachment avoidance—maternal psychological flexibility—adolescent anxiety (indirect effect 1); maternal adult attachment avoidance—mother–adolescent attachment—adolescent anxiety (indirect effect 2); and maternal adult attachment avoidance—maternal psychological flexibility—mother–adolescent attachment—adolescent anxiety (indirect effect 3). The three paths are significant.

The total indirect effect of the chain-mediating effect of maternal psychological flexibility and mother–adolescent attachment on the relationship between maternal adult attachment anxiety and adolescent anxiety is 0.073, accounting for 97% of the total effect. The confidence interval does not contain 0, indicating that maternal psychological flexibility and mother–adolescent attachment have a significant chain-mediating effect on the relationship between maternal adult attachment anxiety and adolescent anxiety.

The chain mediation consists of the following three paths: maternal adult attachment anxiety—maternal psychological flexibility—adolescent anxiety (indirect effect 1); maternal adult attachment anxiety—mother–adolescent attachment—adolescent anxiety (indirect effect 2); and maternal adult attachment anxiety—maternal psychological flexibility—mother–adolescent attachment—adolescent anxiety (indirect effect 3). The three paths are significant. Indirect effect 5 is not significant, while indirect effect 4 and indirect effect 6 are both significant.

### 3.3. Moderated Mediation Effect

Model 87 was used to test the moderated chain-mediating effect, and the results are shown in Table 3 and Table 4.

Table 3 shows that after controlling for gender, age, only-child status, and SSS, in the chain-mediating effect between maternal adult attachment avoidance and adolescent anxiety, under the influence of teacher support, mother–adolescent attachment significantly predicts adolescent anxiety (*β* = −0.143, *p* < 0.01), teacher support does not significantly predict adolescent anxiety (*β* = 0.058, *p* > 0.05), and the interaction between mother–adolescent attachment and teacher support significantly predicts adolescent anxiety (*β* = −0.032, *p* < 0.01). In the chain-mediating effect on the relationship between maternal adult attachment anxiety and adolescent anxiety, under the influence of teacher support, mother–adolescent attachment significantly predicts adolescent anxiety (*β* = −0.143, *p* < 0.01), teacher support does not significantly predict adolescent anxiety (*β* = 0.056, *p* > 0.05), and the interaction between mother–adolescent attachment and teacher support significantly predicts adolescent anxiety (*β* = −0.032, *p* < 0.01).

Table 4 shows that in the chain-mediating effect between maternal adult attachment avoidance and adolescent anxiety, under the influence of peer support, mother–adolescent attachment significantly predicts adolescent anxiety (*β* = −0.138, *p* < 0.01), peer support significantly predicts adolescent anxiety (*β* = 0.096, *p* < 0.05), and the interaction between mother–adolescent attachment and teacher support significantly predicts adolescent anxiety (*β* = −0.036, *p* < 0.01). In the chain-mediating effect between maternal adult attachment anxiety and adolescent anxiety, under the influence of peer support, mother–adolescent attachment significantly predicts adolescent anxiety (*β* = −0.152, *p* < 0.01), peer support does not significantly predict adolescent anxiety (*β* = 0.086, *p* > 0.05), and the interaction between mother–adolescent attachment and peer support significantly predicts adolescent anxiety (*β* = 0.034, *p* < 0.01).

**Figure 2:**
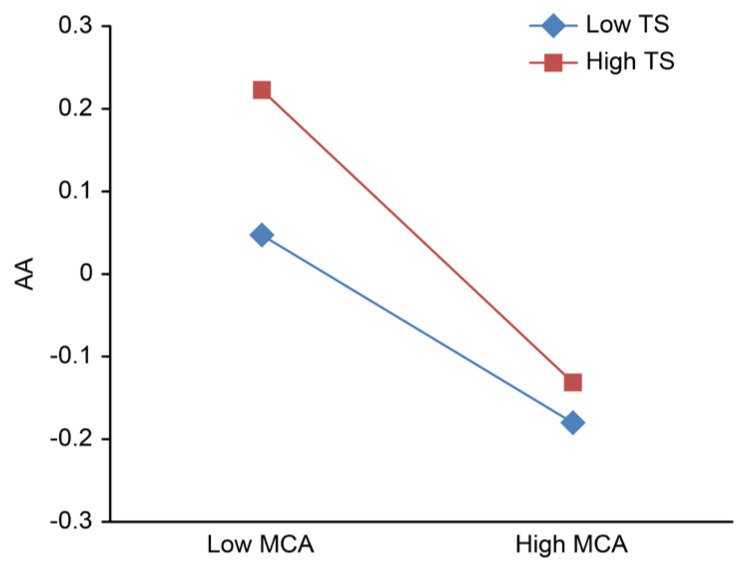
Teacher support adjustment chart a. MCA, mother-adolescent attachment; TS, teacher support; AA, adolescent anxiety.

**Figure 3:**
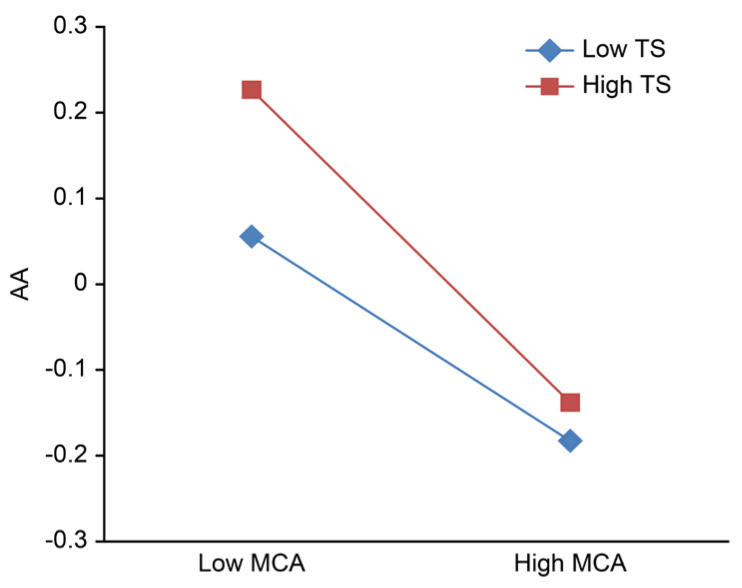
Teacher support adjustment chart b. MCA, mother-adolescent attachment; TS, teacher support; AA, adolescent anxiety.

**Figure 4:**
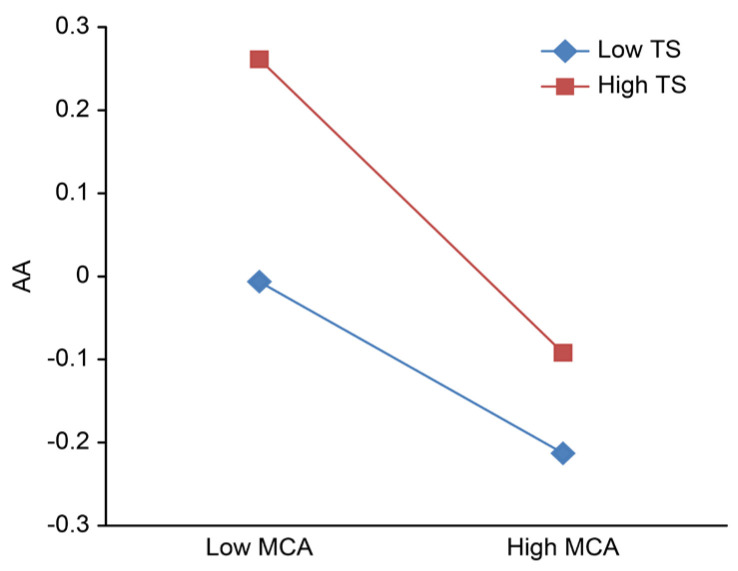
Peer support adjustment chart a. MCA, mother-adolescent attachment; TS, teacher support; AA, adolescent anxiety.

**Figure 5:**
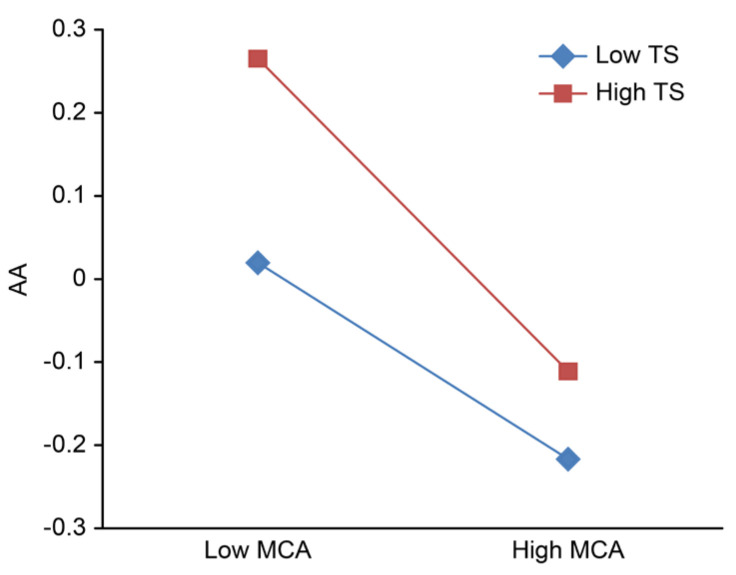
Peer support adjustment chart b. MCA, mother-adolescent attachment; PS, peer support; AA, adolescent anxiety.

To clarify the adjustment trend of the role of teacher support and peer support in the relationship between mother–adolescent attachment and adolescent anxiety, after centralization, high and low groups of teacher support and peer support were defined based on scores one standard deviation below and above the mean; then, the simple slope was calculated, and the adjustment effect was plotted (Figure 2, Figure 3, Figure 4, Figure 5).

Figure 2 and Figure 3 show that regardless of whether the independent variable is maternal adult attachment avoidance (Figure 2) or adult attachment anxiety (Figure 3), the effects of the interaction between mother–adolescent attachment and teacher support on adolescent anxiety are significant. Therefore, a series of simple slope tests was conducted. The results show that when teacher support is high, adolescent anxiety rapidly declines with higher mother–adolescent attachment, while when teacher support is low, adolescent anxiety slowly decreases with more mother–adolescent attachment.

As shown in Figure 4 and Figure 5, regardless of whether the independent variable is maternal adult attachment avoidance (Figure 4) or maternal adult attachment anxiety (Figure 5), the effects of the interaction between mother–adolescent attachment and peer support on adolescent anxiety are significant. Therefore, a series of simple slope tests was conducted. The results show that when peer support is high, adolescent anxiety rapidly decreases with higher mother–adolescent attachment, while when peer support is low, adolescent anxiety slowly decreases with more mother–adolescent attachment.

## 4. Discussion

The present study sought to address a series of questions. Do maternal adult attachment anxiety and maternal adult attachment avoidance directly predict adolescent anxiety? Do maternal psychological flexibility and mother–adolescent attachment mediate the relationship between maternal adult attachment and adolescent anxiety? Do teacher support and peer support moderate the relationship between mother–adolescent attachment and adolescent anxiety? The answers to these questions are important for achieving a better understanding of the causes of anxiety.

The results show that maternal adult attachment avoidance has a direct predictive effect on adolescent anxiety, while maternal adult attachment anxiety has no direct predictive effect on adolescent anxiety, which is consistent with some previous studies [62,63] but inconsistent with others [64]. This may be because mothers with anxious adult attachment often worry about being abandoned, often use overactivation strategies to address intimacy, fear separation from their husbands, and desire excessive intimacy. When interacting with adolescents, these mothers also use excessive activation strategies, and thus, adolescents feel that their mothers’ support is characterized by control and instability, which easily generates conflict between adolescents and their mothers. Mothers with avoidant adult attachment are less sensitive when raising adolescents; lack responsiveness, support, and intimacy; and use deactivation strategies to address intimacy, resulting in adolescents without effective support in stressful situations, leading to anxiety [25].

Our results indicate that maternal psychological flexibility mediates the relationship between maternal adult attachment and adolescent anxiety. This result is consistent with previous studies [62]. The current study further verifies the family systems theory overflow hypothesis. Maternal adult attachment occurs within the marriage subsystem, while maternal psychological flexibility occurs within the mother–adolescent subsystem, and the marriage subsystem overflows to affect the mother–adolescent subsystem [36]. Mothers with anxious or avoidant adult attachment have fewer psychological resources than mothers without such attachment styles when responding to the psychological needs of adolescents and engaging in flexible and effective parenting behaviors. While parenting adolescents, mothers with anxious or avoidant adult attachment are more likely to use control, experience depression, overreact, and employ rigid parenting behaviors [65,66]. Previous research has shown that inflexible parenting behavior is related to adolescent anxiety [67]. Higher maternal psychological flexibility is related to adolescents’ psychological health and other positive psychological qualities, which can help adolescents resist excessive worry under stressful situations [68]. Thus, mothers need to constantly be aware of whether they are affected by potentially problematic adult attachment styles while raising their adolescents.

The results of this study show that mother–adolescent attachment plays a mediating role in maternal adult attachment, which is consistent with the results of previous studies [69]. According to attachment theory, attachment between the mother and father affects the mother’s internal working model, and this internal working model of interpersonal interaction also affects mother–adolescent attachment [70]. Mothers with avoidant adult attachment feel uncomfortable when close to and dependent on others and are more willing to maintain emotional distance from other people. This emotional distancing results in adolescents experiencing less closeness and care in parent–adolescent relationships and more ignorance and indifference [71]. On the one hand, such emotional distancing may directly cause adolescent anxiety; on the other hand, this emotional distancing could also reduce the quality of mother–adolescent attachment to a certain extent and then affect adolescent anxiety [39,72]. Insecure mother–adolescent attachment prevents adolescents from having a stable safe haven in stressful situations and from having strong support, which intensifies their anxiety [72]. The safer the parent–adolescent attachment is, the lower the adolescent anxiety level is [73]. Mothers should provide more care and support to adolescents encountering stressful situations to reduce their anxiety.

Maternal psychological flexibility has a positive effect on mother–adolescent attachment, which is consistent with the results of previous studies [43]. Mothers with high parenting psychological flexibility easily accept negative emotions regarding adolescents and adopt effective and flexible parenting behaviors [29]. When adolescents encounter stressful situations, mothers put aside their excessive worry, anxiety, and avoidance and give adolescents more acceptance and permission. Flexible and effective maternal parenting behavior provides adolescents with an effective safe haven, helps them form a safe attachment, and gives them a full sense of security to explore the unknown world [40], which effectively buffers anxiety among adolescents [74].

High teacher support and peer support can enhance the resistance of mother–adolescent attachment to adolescent anxiety. The two protective factors at home and school interact with each other to enhance individual development, suggesting that adolescents can feel greater warmth from their mothers when they have good teacher–student relationships and peer relationships. According to Bronfenbrenner’s microsystem of social ecosystem theory, home and school are the environments where adolescents have the closest contacts. School is the place where adolescents have the closest contact with people other than their families. Teachers and peers are adolescents’ main contacts. Teacher support and peer support also have important impacts on adolescent anxiety [59]. This result further validates Bowlby’s view that secure mother–adolescent attachment is the main factor influencing adolescent anxiety [75]. This study also found that mother–adolescent attachment has a stronger resistance to adolescent anxiety under high teacher support than under high peer support, indicating that compared with peers, teachers have a greater effect on reducing adolescent anxiety. This finding suggests that in interactions between teachers and adolescents, teachers should provide adolescents with more warmth and care so that adolescents can feel warmth and support from teachers, which plays an important role in reducing adolescents’ anxiety.

### 4.1. Limitations and Future Research Directions

Several limitations may affect the generalizability of the findings of this study. First, this study adopted the method of convenience sampling and drew its subjects from only one province in China rather than all provinces; therefore, in order to obtain results that are more representative of Chinese youth in general, it would be appropriate for future studies to adopt the method of stratified sampling in different provinces. Even then, the findings would not be generalizable outside the Chinese population; for example, they might not be representative of white or Hispanic populations. Therefore, future studies of adolescents in different countries should also be considered. Second, this study used a cross-sectional research design instead of a longitudinal research design. A longitudinal design could be more fruitful in further investigations examining the causal sequence and identifying trajectories and consequences. Furthermore, other variables, such as genetic factors and history of abuse, may influence anxiety among adolescents. Further studies may consider including these variables to control for their potential influences. Finally, although mothers are the main caregivers in most families, fathers also contribute to children’s emotional and behavioral outcomes; thus, further research should examine paternal adult attachment to better investigate the effects of adolescent anxiety.

### 4.2. Research Implications

Despite these limitations, our research provides a multiple mediation model of the relationship between maternal adult attachment and adolescent anxiety through maternal psychological flexibility and mother–adolescent attachment in a Chinese sample and explores the moderating roles of teacher support and peer support in the relationship between mother–adolescent attachment and adolescent anxiety. This research also extends the literature through its use of cross-informant ratings within a Chinese context with both mother and adolescent self-reports. Moreover, the simultaneous investigation of maternal adult attachment styles (avoidant attachment and anxious attachment) and teacher support in the current study extends the existing literature by showing that maternal adult attachment avoidance demonstrates more of a relationship with adolescent anxiety than maternal adult attachment anxiety.

Teacher support is stronger than peer support in regulating the relationship between mother–adolescent attachment and adolescent anxiety. From a prevention standpoint, it is essential to encourage Chinese mothers to reduce their adult attachment avoidance and enhance their psychological flexibility, which may be a key intervention for mitigating the development of adolescent anxiety.

## 5. Conclusions

The present study adds to the growing literature concerning the association between maternal adult attachment and adolescent anxiety. The findings reveal the chain-mediating effect of maternal psychological flexibility and mother–adolescent attachment on the relationship between maternal adult attachment and adolescent anxiety, particularly in the Chinese context. Based on social–ecological systems theory and family systems theory, researchers and practitioners should emphasize the importance of mothers, teachers, and peers in adolescent anxiety, particularly in terms of mothers’ avoidance of adult attachment and teacher support. A key message is that the combination of mothers’ positive responses to adolescents and teachers’ support of adolescents has the strongest effect on reducing adolescent anxiety.

## Figures and Tables

**Table 1:** Pearson correlations and descriptive statistics of the main study variables (N = 1139).

	*M*	*SD*	1	2	3	4	5	6	7	8	9	10
1. MAA1	3.22	0.88	1									
2. MAA2	3.30	1.15	0.455 **	1								
3. MPF	4.57	0.96	−0.515 **	−0.644 **	1							
4. MCA	3.91	0.65	−0.233 **	−0.210 **	0.302 **	1						
5. AA	2.13	0.42	0.299 **	0.224 **	−0.335 **	−0.601 **	1					
6. TS	4.97	1.32	−0.067 *	0.068 *	0.096 **	0.378 **	−0.403 **	1				
7. PS	5.14	1.19	−0.167 **	−0.044	0.165 **	0.303 **	−0.314 **	0.625 **	1			
8. Gender	-	-	0.043	−0.034	−0.005	0.034	0.044	−0.039	−0.032	1		
9. Age	-	-	−0.009	0.004	−0.009	−0.075 *	0.062 *	−0.121	−0.034	−0.048	1	
10. ME	-	-	−0.205	−0.235	0.232 **	0.071 *	−0.065 *	−0.057	0.056	0.024	−0.069 *	1

Note. MAA1, maternal adult attachment (avoidant attachment); MAA2, maternal adult attachment (anxious attachment); MPF, maternal psychological flexibility; MCA, mother–adolescent attachment; AA, adolescent anxiety; TS, teacher support; PS, peer support, ME mother’s education. * *p* < 0.05. ** *p* < 0.01.

**Table 2:** Bootstrap confidence interval and effect size of the indirect effect model (N = 1139).

Indirect Effect	Effect Value	Effect Amount (%)	95% CI	
			Lower	Upper
MAA1				
MAA1 → MPF → AA	0.031	20	0.014	0.048
MAA1 → MCA → AA	0.032	19	0.013	0.050
MAA1 → MPF → MCA → AA	0.035	21	0.025	0.046
Total Mediating Effect	0.097	60	0.070	0.122
MAA2				
MAA2 → MPF → AA	0.043	52	0.025	0.062
MAA2 → MCA → AA	0.006	1	−0.010	0.023
MAA2 → MPF → MCA → AA	0.040	44	0.029	0.052
Total Mediating Effect	0.089	97	0.067	0.113

Note. MAA1, maternal adult attachment (avoidant attachment); MAA2, maternal adult attachment (anxious attachment); MPF, maternal psychological flexibility; MCA, mother–adolescent attachment; AA, adolescent anxiety.

**Table 3:** Test of the moderating effect of teacher support (N = 1139).

	Model (MAA1–AA)			Mode2 (MAA2–AA)		
	*SE*	*β*	*t*	*SE*	*β*	*t*
Age	0.006	0.004	0.631	0.006	0.004	0.543
ME	0.007	−0.008	−1.162	0.007	−0.007	−1.040
MCA	0.051	−0.143 **	−2.832	0.051	−0.149 **	−2.917
TS	0.040	0.058	1.461	0.040	0.056	1.379
MCA × TS	0.010	−0.032 **	−3.177	0.010	−0.032 **	−3.127
R^2^		0.441			0.432	
F		127.210			122.651	

Note. SJJ, adolescents compared with residents in their province; XJJ, adolescents compared with students in their school; MCA, mother–adolescent attachment; TS, teacher support; MAA1, maternal adult attachment (avoidant attachment); MAA2, maternal adult attachment (anxious attachment); AA, adolescent anxiety. ** *p* < 0.01.

**Table 4:** Test of the moderating effect of peer support (N = 1139).

	Model (MAA1—AA)			Mode2 (MAA2—AA)		
	*SE*	*β*	*t*	*SE*	*β*	*t*
SJJ	0.006		0.425	0.007	0.002	0.296
XJJ	0.007	−0.012	−1.705	0.07	−0.011	−1.523
MCA	0.059	−0.138 **	−2.348	0.059	−0.152 **	−2.561
PS	0.044	0.096 *	2.201	0.044	0.086	1.954
MCA × PS	0.011	−0.036 **	−3.216	0.011	−0.034 **	−3.027
R^2^		0.419			0.410	
F		116.542			122.485	

Note. SJJ, adolescents compared with residents in their province; XJJ, adolescents compared with students in their school; MCA, mother–adolescent attachment; PS, peer support; MAA1, maternal adult attachment (avoidant attachment); MAA2, maternal adult attachment (anxious attachment); AA, adolescent anxiety * *p* < 0.05. ** *p* < 0.01.

## Data Availability

The data presented in this study are available on request from the corresponding author.
